# Anticalin N- or C-Terminal on a Monoclonal Antibody Affects Both Production and In Vitro Functionality

**DOI:** 10.3390/antib11030054

**Published:** 2022-08-22

**Authors:** Nicolas Aubrey, Valérie Gouilleux-Gruart, Christine Dhommée, Julie Mariot, Fanny Boursin, Nicolas Albrecht, Cécile Bergua, Cécile Croix, Mäelle Gilotin, Eloi Haudebourg, Catherine Horiot, Laetitia Matthias, Caroline Mouline, Laurie Lajoie, Audrey Munos, Gilles Ferry, Marie-Claude Viaud-Massuard, Gilles Thibault, Florence Velge-Roussel

**Affiliations:** 1 ISP UMR 1282, INRA, Team BioMAP, University of Tours, 31 Avenue Monge, 37200 Tours, France; 2 GICC EA7501, Team FRAME, University of Tours, 10 boulevard Tonnellé, 37032 Tours, France; 3 GICC EA7501, Team IMT, University of Tours, 10 boulevard Tonnellé, 37032 Tours, France; 4 Institut du Médicament de Tours, BIO3, 15 rue du plat d’étain, 37000 Tours, France; 5 Chemistry Manufacturing and Control—Biologics, Institut de Recherches SERVIER, 78290 Croissy-sur-Seine, France

**Keywords:** monoclonal antibody, bispecific antibody, anticalin, Fc gamma receptors, FcRn, CD16, CD32, ADCC, CDC

## Abstract

Bispecific antibodies (BsAbs) represent an important advance in innovative therapeutic strategies. Among the countless formats of BsAbs, fusion with molecules such as anticalins linked to a monoclonal antibody (mAb), represents an easy and low-cost way to obtain innovative molecules. We fused an anticalin against human fibronectin to a molecule biosimilar to trastuzumab (H0) or rituximab (R0), in four different positions, two on the N terminal region of heavy or light chains and two on the C terminal region. The eight BsAbs (H family (HF) 1 to 4 and R family (RF) 1 to 4) were produced and their affinity parameters and functional properties evaluated. The presence of anticalin did not change the glycosylation of the BsAb, shape or yield. The antigenic recognition of each BsAb family, Her2 for HF1 to 4 and CD20 for RF1 to 4, was slightly decreased (HF) or absent (RF) for the anticalin N-terminal in the light chain position. The anticalin recognition of FN was slightly decreased for the HF family, but a dramatic decrease was observed for RF members with lowest affinity for RF1. Moreover, functional properties of Abs, such as CD16 activation of NK, CD32-dependent phagocytosis and FcRn transcytosis, confirmed that this anticalin position leads to less efficient BsAbs, more so for RF than HF molecules. Nevertheless, all BsAbs demonstrated affinities for CD16, CD32 and FcRn, which suggests that more than affinity for FcRs is needed for a functioning antibody. Our strategy using anticalin and Abs allows for rapid generation of BsAbs, but as suggested by our results, some positions of anticalins on Abs result in less functionality.

## 1. Introduction

Bispecific antibodies (BsAb), which combine specificities of two antibodies, have become the most promising classes of therapeutic antibodies. As referenced by [1], there is a limitless domain of innovative design for these molecules because of molecular engineering involving the association of different types of binding molecules, the combination of two different heavy chain/light chain variable domains (VH/VL), but also molecules such as the single-chain variable fragment (scFv) and the camelid heavy chain variable domain (V_H_H) associated together or with an antibody (Ab) shape. The unique ability to tether two antigen specificities into a single protein structure has allowed for new therapeutic strategies and opened a new field of fundamental knowledge in immunology. The only limitation is the structural protein biological laws that determine the optimal 3D shape of the functional bispecific Ab. Such molecule configurations that already have US Food and Drug Administration or European Medicines Agency agreement demonstrated the possibility to target both tumors and T cells [2], bridging two enzymes to offset the missing catalytic factor [3] or modulating the immune response by capturing cytokines that are both a cause and an amplifying factor of inflammation mechanisms in autoimmune diseases [4]. According to the number of BsAbs patented or in development [5], their interest is validated and their growing development will support new therapeutic strategies and a significant increase in knowledge of immunology.

In addition to BsAbs that could be said to be conventional, many other molecules have binding properties that could be judiciously used in combination, or not, with Abs [6]. These molecules are able to bind to other molecules, such as VHH lipocalins, adnectins, aptamers, or DARPins with a non-immunoglobulin scaffold for the last four families [7,8]. All of these molecules together can be considered as “engineered affinity proteins” and could be a breeding ground for new antigenic specificities or new formats such as lipocalins. Lipocalins are a group of circulating, abundant, extracellular proteins [9]. In humans, 12 different lipocalins have been identified and serve to transport hydrophobic or chemical molecules in blood and lymph. These molecules are characterized by a structurally conserved rigid α-barrel structure and four flexible loops. In particular, the anticalins have been reprogrammed from lipocalins by protein engineering based on their structural similarity with the combining site of immunoglobulins (Igs). Some companies have already understood the potential of merging anticalins with monoclonal antibodies (mAbs). Pieris Pharmaceuticals, has a huge pipeline to develop molecules against cancer, asthma and COVID-19 pathogens. A large number of anticalins have been developed by combinatorial protein engineering; they present various antigen specificities, such as against vascular endothelial growth factor A [10] or the plant digoxigenin [6]. With their small size and high affinity, these molecules are suitable tools for diagnosis [11] and new therapeutic developments [12].

Regarding the potential value of merging an anticalin with an antibody shape, we attempted to use anticalins to design a BsAb with a new shape. Such molecules were readily produced in mammalian expression systems and exhibited favorable biophysical properties [13]. We chose the well-known trastuzumab (TTZ) and rituximab (RTX) mAbs and connected them at C- or N-terminal of each light (L) and heavy (H) chain, an anticalin molecule specific for the EB domain of human fibronectin (Fn) and the anticalin N7A (ACFn) [14]. A priori, these positions may have different effects on mAb functional properties. Indeed, the anticalin linked on the N-terminal of both L and H chains (i.e., in the vicinity of the paratope) could strongly influence the antigen binding of the mAb. In the same way, the anticalin linking the C-terminal of H or L chains (i.e., at the end of the Fc or in the vicinity of the hinge region) could affect Fc-dependent properties. Finally, whatever the position, the linking of the anticalin could affect the anticalin functional properties, especially its ability to bind to its cognate ligand. Therefore, all these positions were evaluated, which led to building eight different molecules (four for each therapeutic Ab). We examined the biophysical properties of the novel molecules, such as their thermostability and characterized their higher-order structure, as well as binding to their target antigens. Particular attention was paid to their capacity to bind to FcγRs such as CD16A, CD32A and FcRn because of the functional properties that result from it, in particular the ability of the hybrid molecules (BsAbs) to induce effector mechanisms such as functional responses of natural killer (NK) cells, CD32A-dependent phagocytosis by macrophages and FcRn-dependent transcytosis. In summary, this work seeks to understand the best position for an anticalin to fuse to an Ab format with respect to the functionality of the BsAb so formed.

## 2. Materials and Methods

### 2.1. Design and Construction

The anticalin selected in this study was the neutrophil gelatinase-associated lipocalin protein N7A [14]. The sequence from the protein data bank is QDSTSDLPAPPLKVPLQQNFQDNQFHGKWVVGKAGNHDLREDKDPRMQATYELKEDKSYNVTNVRFVHKKCNYRIWTFVPGSQPGEFLGNIKSWPGLTSWLVRVVSNYNQHAMVFFKRVYQNRELFEITLYGRTKELTNELKENFIRFSKSLGLPENHIVFPVPIDQCIDG. For anticalin purification, a twin strep-tag was added at the C-terminal position (SAWSHPQFEKGG-GSGGGSGGSAWSHPQFEK). The corresponding gene was synthesized and cloned in pcDNA3.4 by using GeneArt (Thermo Fisher Scientific, Waltham, MA, USA). The heavy (H) and light (L) chains of TTZ (H0) and RTX (R0) were synthesized and cloned in pcDNA3.4 by using GeneArt (Thermo Fisher Scientific, Waltham, MA, USA). We obtained four plasmids: pcDNA3.4-HH, pcDNA3.4-HL, pcDNA3.4-RH, pcDNA3.4-RL for H and H chains of TTZ and H and L chains of RTX, respectively. Anticalin was fused on the H or L chain of each antibody at the N-terminal or C-terminal position with the linker “GGGGS GGGGS GGGGS”. For generating the plasmid pcDNA3.4-fusion, the golden gate technique was used (New England Biolabs). The genes were amplified by PCR with suitable primers, and ligations involved using BsaI-HF v2 (New England Biolabs). Eight plasmids were obtained: pcDNA3.4-ACFn-HH, pcDNA3.4-HH-ACFn, pcDNA3.4-ACFn-HL, pcDNA3.4-HL-ACFn and pcDNA3.4-ACFn-RH, pcDNA3.4-RH-ACFn, pcDNA3.4-ACFn-RL, pcDNA3.4-RL-ACFn. TG1 chemically competent bacteria were transformed with the newly-formed plasmids and constructions were validated by sequencing.

### 2.2. Expression and Purification

All proteins were produced by transient transfection of ExpiCHO cells with the max titer protocol as described in the Thermo Fisher manufacturer’s protocols. Briefly, cells were grown in ExpiCHO Expression Medium and maintained in a humidified incubator with 8% CO_2_ at 37 °C with shaking. Before the transfection (day-1), cell concentration was adjusted to 4.10^6^ viable cells/mL and incubated overnight in culture growth conditions. On the next day (day 0), cell culture was diluted to 6.10^6^ cells/mL and ExpiFectamine CHO–DNA complexes were added slowly to a flask with gently swirling. For Abs, the proportion of H and L chains were determined with a 1:3 ratio. The expression enhancer was added at 18 h to 22 h post-transfection, and the flask was placed at 32 °C with 5% CO_2_. An additional expression feed was added on day 5, and cells were harvested at about day 10 post-transfection. Cell viability was measured by using CytoSMART (Corning) and cells were centrifuged at 10,000× *g* for 10 min. Clarified supernatants were stored at −20 °C until purification.

For purification of antibodies, samples were deep-frozen, centrifuged at 10,000× *g* for 20 min and passed over a 0.22-µm filter. All supernatants were passed over the HiTrap Protein A HP column (Cytiva, 17040301) in the ÄKTA pure protein purification system and equilibrated with phosphate buffered saline (PBS) buffer (2.7 mM KCl, 0.10 M NaCl, 2 mM KH2PO4, 8 mM Na2HPO4, pH 7.4). The column was washed with four column volumes of PBS, and proteins were eluted with 0.1 M citrate, pH 3.

For purification of anticalin, samples were deep-frozen and dialyzed overnight at +4 °C in binding buffer (20 mM NaH_2_PO_4_, 280 mM NaCl, 6 mM KCl, pH 7.4), centrifuged at 1000× *g* for 20 min and filtered with a 0.22-µm filter. All supernatants were passed over a StreTrap HP column (Cytiva, 28907546) in the ÄKTA pure protein purification system and equilibrated with binding buffer. The column was washed with four column volumes of PBS-ST, and protein was eluted with elution buffer (2.5 mM desthiobiotin in binding buffer).

The proteins were desalted through the HiPrep 26/10 desalting column (Cytiva Life sciences, Marlborough, MA, USA) in PBS buffer. The concentration was determined with a UV detector at 280 nm. Protein molecular mass and molar extinction coefficient data were generated by using the Protparam tool (http://web.expasy.org/protparam/ (accessed on 1 April 2018)).

Proteins were concentrated by centrifugation using Amicon Ultra (Merk Millipore, Molsheim, France) 30 kD until a molarity of about 30 µM was obtained for Abs and fusions and with Amico Ultra 10 kD (Merk Millipore, Molsheim, France) until a molarity of about 10 µM was obtained for anticalin. Proteins were filtered with a 0.22-µm filter and stored at +4 °C before analysis. For long-term storage, proteins were kept at −20 °C.

### 2.3. Biophysical Characterization

#### 2.3.1. SDS-PAGE

The integrity of all purified proteins was assessed by SDS-PAGE on homogeneous 10% polyacrylamide gel under reducing or non-reducing conditions. Samples were all loaded at 1 µg for anticalin, 3.8 µg for H0 and R0 and 5 µg for mAbs or BsAbs, for Coomassie blue staining. ProSieve QuadColor Protein Markers (Lonza, Fribourg, Switzerland) were used. For anticalin, after migration in a SDS-PAGE gel, proteins were transferred onto nitrocellulose membranes (Invitrogen, Carlsbad, CA, USA), which were blocked with 5% non-fat milk diluted in TNT (15 mM Tris–HCl, 140 mM NaCl, 0.05% Tween 20) overnight at +4 °C with shaking, then incubated for 1 h 30 min at room temperature with Strep-Tactin AP Conjugate (IBA-Lifesciences, Gottingen, Germany) diluted 1:1000 in 5% milk-TNT. After washes with TNT, alkaline phosphatase activity was detected by using BCIP/NBT substrate (Promega, Chicago, IL, USA).

#### 2.3.2. Size-Exclusion Chromatography-High Performance Liquid Chromatography (SEC-HPLC)

The purified proteins were analyzed by *SEC-HPLC* on a Superdex 200 Increase 10/300 GL column (Cytiva Life sciences) with an Akta purifier. The column was loaded with 100 µL of 5 µM for each protein (20 µg for anticalin, 109 µg for H0, 142 µg for HF1 to HF4, 108 µg for R0 and 141 µg for RF1 to RF4) and was eluted with PBS at 0.4 mL/min. Protein was detected with a UV detector at 280 nm. Volume of elution and percentage of peak area were determined for analysis of monomeric antibodies.

#### 2.3.3. Thermal and Aggregation Analysis

Prometheus NT.48 was used to measure the thermal unfolding profiles of proteins by differential scanning fluorimetry experiments (Prometheus NT.48, NanoTemper, Munich, Germany). All samples were used at a final concentration of 10 µM and loaded into high-sensitivity capillaries (Nanotemper, Munich, Germany). The protein unfolding process was subjected to a thermal ramp (20–95 °C, 1 °C/min). Data analysis involved using Prometheus PR ThermControl software (NT. 48, NanoTemper, Munich, Germany). The Tm value was determined by fitting the tryptophan 350/330 nm fluorescence emission ratio using a polynomial function in which the maximum slope is indicated by the peak of its first derivative.

#### 2.3.4. Mass Spectrometry (MS)

Characterization of Abs was performed by high-resolution MS using an Acquity UPLC H-Class system coupled with a Vion IMS QT mass spectrometer, both from Waters (Waters, Wilmslow, UK). Before MS analysis, 800 ng Ab was injected onto a XBridge Protein BEH C4 2.1 × 30 mm, 1.7-μm column heated to 90 °C. A desalting step was carried out with 95% solvent A (H_2_O + 0.1% formic acid) and 5% solvent B (acetonitrile + 0.1% formic acid) during 2 min at 0.5 mL/min, with the flow diverted to waste. After that, a 3 min gradient from 5% to 50% of solvent B then a gradient from 50% to 90% of solvent B was applied at a 0.4 mL/min flow rate to elute the sample with the flow diverted to MS. MS data were acquired in positive ionization mode with an ESI source over a 500- to 4000-*m*/*z* window with 1-Hz scan. Voltage capillary was set to 2.5 kV; desolvation temperature and source temperature to 600 °C and 120 °C, respectively; and cone voltage 150 V. The results were processed by using Waters (Milford, MA, USA), software UNIFI v1.9.4 and the MaxEnt1 algorithm was used for deconvolution.

#### 2.3.5. Interferometry

Affinities of constructs were measured by biolayer interferometry (BLI) with an RED96 Octet system (ForteBio Sartorius, Hamburg, Germany) at 25 °C with 0.1 M phosphate buffer, 150 mM NaCl, and 0.05% Tween 20 at pH 7.4 as running buffer. For H0 and HF1-4 BsAbs, recombinant Her-2 (12.5 μg/mL) was immobilized on penta-His biosensors, then H0 or BsAbs were associated (300-s concentrations of 400, 200, 100, 50 and 0 nM). Then dissociated (300 s) before regeneration with glycine 10 mM at pH 1.5. For ACFn, RF1-4 and HF1-4 BsAbs, recombinant fibronectin (FN) (5–20 μg/mL) was immobilized on penta-His biosensors, and the variants were associated (600-s concentrations of 200, 100, 50, 25 and 0 nM), then dissociated (600 s) before regeneration (glycine 10 mM, pH 1.5). For data analysis, the 0 nM wells were used as a reference, the y-axis was aligned on the last second of the baseline, the interstep correction was based on dissociation, and the fitting followed a 1/1 model during the first 10 s of dissociation. Resulting K_D_ (affinity) values were determined from the aligned and referenced sensograms in Data Analysis HT10.0 software with reported values as the mean of ≥5 independent runs.

### 2.4. Affinity Measurement

#### 2.4.1. Affinity to CD16

Human CD16A (FcγRIIIA-V158)-transduced NK92 (NK92-CD16A) cells (kindly provided by Dr. B. Clemenceau, INSERM CR 1232 CRCINA, Nantes, France) were grown in RPMI 1640 culture medium (Sigma-Aldrich, St. Louis, MO, USA) supplemented with 10% heat-inactivated fetal bovine serum (FBS; Dominique Dutscher, Brumath, France), 1 mM sodium pyruvate (Invitrogen, Carlsbad, CA, USA); 50 units/mL penicillin (Sigma-Aldrich, St. Louis, MO, USA); and 50 μg/mL streptomycin (Sigma-Aldrich, St. Louis, MO, USA) and 100 UI/mL IL-2 (Proleukin) (Chiron Corp, Emeryville, CA, USA.).

NK92 hCD16V cells were used to analyze CD16A binding in competition experiments. NK92 hCD16V cells were first incubated with increasing concentrations of the mAbs or BsAbs of interest for 30 min at 4 °C. Then NK92 hCD16V cells were incubated with anti-CD16 antibody (3G8 clone) labeled with FITC (Beckman Coulter, Brea, CA, USA). The mean fluorescence intensity (MFI) was measured by flow cytometry to determine the percentage of inhibition binding on NK92 hCD16V cells. Flow cytometry was performed on a Cytoflex S flow Cytometer (Beckman Coulter, Brea, CA, USA) with FlowLogic software 7 (Miltenyi Biotec, Paris, France).

Results are expressed as a percentage of anti-CD16-FITC inhibition binding. The point with anti-CD16-FITC alone was considered 100% binding. Cells stained by isotype antibody-labeled FITC (Beckman Coulter, Brea, CA, USA) were used to evaluate Ab non-specific binding. Results were calculated as a percentage of the ratio of anti-CD16-FITC minus isotype-FITC and mAb (X) MFI.

#### 2.4.2. Affinity to CD32

The THP1 cell line was maintained in RPMI 1640 culture medium (Sigma-Aldrich, St. Louis, MO, USA), supplemented with 10% heat-inactivated FBS (Dominique Dutscher, Brumath, France), 2 mM L-glutamine (Sigma-Aldrich, St. Louis, MO, USA), 1 mM sodium pyruvate (Invitrogen, Carlsbad, CA, USA), 500 units of 0.5 mg/mL penicillin-streptomycin (Sigma-Aldrich, St. Louis, MO, USA) and 50 µM 2-mercaptoethanol (Serva, Heidelberg, Germany). THP1 cells were first incubated with increasing concentrations of the mAbs or BsmAbs of interest for 30 min at 4 °C, then incubated with anti-CD32 antibody (clone IV.3) FITC-labeled (Stemcell, Vancouver, BC, Canada). MFI was measured by flow cytometry to determine the percentage of inhibition binding on THP1 cells. Flow cytometry was performed on a Cytoflex S flow Cytometer (Beckman Coulter, Brea, CA, USA) with FlowLogic software (Miletnyi Biotec, Paris, France). Results are expressed as the percentage of anti-CD32-FITC inhibition binding. The point with anti-CD32-FITC alone was considered 100% binding. Cells stained by isotype antibody FITC-labelled (Stemcell, Vancouver, BC, Canada) were used to evaluate Ab non-specific binding. Results were calculated as a percentage of the ratio of anti-CD32-FITC minus isotype-FITC and mAb (X) MFI.

#### 2.4.3. Affinity to FcRn

JurkatΔFcRn cells were obtained by stable transfection of the Jurkat cell line (ATCCrg TIB-152TM) with a plasmid containing the human FcRn (hFcRn) sequence deleted from 99 nt corresponding to the 33 aa of the C-terminal protein. The Jurkat cell line was maintained in RPMI 1640 culture medium (Sigma-Aldrich, St. Louis, MO, USA) supplemented with 10% heat-inactivated FBS (Dominique Dutscher, Brumath, France), 2 mM L-glutamine (Sigma-Aldrich, St. Louis, MO, USA), 500 units of 0.5 mg/mL penicillin-streptomycin (Sigma-Aldrich, St. Louis, MO, USA), and 1 mg/mL G418 (Thermofisher Scientific, Waltham, MA, USA) was added for JurkatΔFcRn cells.

RTX was conjugated to Alexa Fluor 488 fluorescent dye (rituximab^AF488^) by using the Ab labeling kit (Thermofisher Scientific, Waltham, MA, USA), according to the manufacturer’s instructions. Rituximab^AF488^ was used at 4 μg/mL in competition with unlabeled RTX (R0), TTZ (H0), or the BsmAbs of interest at 0.04- to 30-µM concentrations to evaluate FcRn binding. Jurkat and JurkatΔFcRn cells were mixed in a 1:1 ratio. In a microtitration 96-well plate, 4 × 10^4^ cells/well were suspended in HBSS (PAN Biotech, Aidenbach, Germany) adjusted to pH 6 with MES (Sigma-Aldrich, St. Louis, MO, USA) and incubated 30 min at 4 °C with rituximab^AF488^ and the different mAbs or BsAbs at increased concentrations. MFI was measured by flow cytometry (Cytoflex S, Beckman Coulter, CA, USA). FlowLogic software (MiltenyiBiotec, Paris, France) was used for analysis. Results are expressed as the percentage of rituximab^AF488^ inhibition binding. The point with rituximab^AF488^ alone was considered 100% binding. Untransfected Jurkat cells (WT) were used to evaluate Ab non-specific binding. Results were calculated as a percentage of the ratio of mAb (X) MFI JurkatΔFcRn/JurkatWT and rituximab^AF488^ MFI JurkatΔFcRn/JurkatWT.

#### 2.4.4. Affinity to Target Antigens

Competition assays were set up with fluorescent RTX on Daudi cells expressing CD20 and with fluorescent TTZ on SK-BR-3 cells positive for Her2. Rituximab^AF488^ (see Section 2.4.3) was used at 1 μg/mL in competition with unlabeled R0 or RF1-4 at 0.2- to 250-fold rituximab^AF488^ concentrations. TTZ was conjugated to Alexa Fluor 488 fluorescent dye (trastuzumab^AF488^) by using the Ab labelling kit (Thermofisher Scientific, Waltham, MA, USA), according to the manufacturer’s instructions. Trastuzumab^AF488^ was used at 0.1 ug/mL in competition with unlabeled H0 or BsAbsat 0.2- to 250-fold trastuzumab^AF488^ concentration. Daudi cells (4.10^4^ cells) and SK-BR-3 cells (4.10^4^ cells) were resuspended in culture medium and incubated with rituximab^AF488^ and trastuzumab^AF488^ respectively and/or the different mAbs at various concentrations.

After 30 min at 4 °C, MFI was evaluated by flow cytometry (Cytoflex S) and analyzed with FlowLogic. Results are expressed as the MFI of rituximab^AF488^ binding inhibition on Daudi cells or trastuzumab^AF488^ binding inhibition on SK-BR-3 cells. Rituximab^AF488^ or trastuzumab^AF488^ alone were considered 100% binding for determining the percentage of inhibition binding.

### 2.5. Functional Analyses

#### 2.5.1. Cell Culture

The Daudi cell line (ATCC Cat#CCL-213) and SK-BR-3 cell line (ATCC Cat#HTB-30) were used for experiments targeting CD20 and HER2, respectively. Daudi cells were cultured in RPMI-1640 (Sigma-Aldrich, St. Louis, MO, USA) containing 10% FBS (Dominique Dutscher, France), 100 U/mL penicillin and 100 µg/mL streptomycin (Sigma-Aldrich, St. Louis, MO, USA). SK-BR-3 cells were maintained in DMEM Glutamax culture medium (Thermo Scientific, Waltham, MA, USA) containing 10% FBS (Dominique Dutscher, France), 100 U/mL penicillin and 100 µg/mL streptomycin (Sigma-Aldrich, St. Louis, MO, USA).

MDCKII cells expressing human FcRn [15] were maintained in RPMI 1640 culture medium (Sigma-Aldrich, St. Louis, MO, USA) supplemented with 10% heat-inactivated FBS (Dominique Dutscher, Brumath, France), 2 mM l-glutamine (Sigma-Aldrich, St. Louis, MO, USA), 500 units of 0.5 mg/mL penicillin-streptomycin (Sigma-Aldrich, St. Louis, MO, USA) and 4 mg/mL G418 (Thermofisher Scientific, Waltham, MA, USA).

#### 2.5.2. Phagocytosis

To prepare peripheral blood mononuclear cells (PBMCs), whole blood was diluted with PBS. Density gradient separation of blood involved using Lymphoprep (Eurobio, 91940 Les Ulis, France). Tubes were centrifuged at 450× *g* for 25 min at 25 °C degrees, then cell layers (buffy coat) were immediately collected and transferred to 50 mL conical tubes, resuspended with PBS and centrifuged at 300× *g* for 10 min at 25 °C.

CD14^+^ cells were sorted by positive selection from PBMCs by using CD14 MicroBeads and a MS Column, according to the manufacturer recommendations (Miltenyi Biotec, Germany). CD14^+^ cells were cultured in X-VIVO culture medium (Thermo Scientific, Waltham, MA, USA) supplemented with M-CSF at 60 ng/mL (Miltenyi Biotec, Paris, France). Medium with added M-CMF was changed 3 days later for differentiation to macrophages. At day 6, SKBR3 or Daudi cells stained with CFDA-SE (Stemcell, Vancouver, BC, Canada) were incubated with CD14+ cells differentiated in a 1:1 ratio (E:T) in the presence of the cytokine interleukin 10 (IL-10; 1/500; Stemcell, Vancouver, BC, Canada), with mAbs at 10 µg/mL for 3 h at 37 °C, 5% CO_2_. Then cells were stained with viability-fixable violet dead cell marker (ThermoFisher, Waltham, MA, USA) and anti-CD64 APC-Vio770-labelled antibody (Miltenyi Biotec, Bergisch Gladbach, Germany) for 20 min at 4 °C. The MFI was analyzed by FCM on at least 5 × 10^4^ cells.

#### 2.5.3. Transcytosis

Transcytosis experiments were performed as described (Ternant et al. 2016), with the following modifications. Briefly, MDCKII cells expressing human FcRn (7.5.10^4^ cells/well, 3 wells for each condition) were seeded in 96-well Transwell plates (0.4 µm, Merck Milliport, Burlington, MA, USA) with 75 µL and 250 µL growth medium in the inner and outer chambers, respectively. After 2 days, rituximab^AF488^ (see Section 2.4.3) was introduced in the outer chambers at 20 μg/mL in the presence of unlabeled RTX or TTZ or the mAbs or BsAbs to be tested (15.63–1000 µg/mL) in HBSS– 0.67% gelatin (Sigma-Aldrich, St. Louis, MO, USA) adjusted to pH 6 with MES (Sigma-Aldrich, St. Louis, MO, USA) for 2 h at 37 °C, 5% CO_2_. Then, supernatants were removed from the apical pole and transferred into black 384-well plates (Dominique Dutscher, Brumath, France). Fluorescence was measured by microplate reader (Mithras LB940, Berthold, Germany). Wells containing rituximab^AF488^ alone were considered 100% mAb transcytosis. Results (mean of triplicate) are expressed as inhibition of rituximab^AF488^ transcytosis.

#### 2.5.4. CD16A-Dependent Functional Responses

To evaluate CD16A-dependent functional responses, NK92 hCD16V (see Section 2.4.1) effector cells were incubated with target cells. NK92 hCD16V (5 × 10^4^ cells) were incubated for 4 h with Daudi or SK-BR-3 cells (E:T 1:2) in the presence of 10 µg/mL of mAbs or BsmAbs at 37 °C, anti-CD107a mAb and 0.1 µg/mL BD GolgiPlug containing Brefeldin A (BD Biosciences, San Jose, CA, USA) at 37 °C in 5% CO2 humidified air. Cells were then stained with anti-CD16 and anti-CD56 Abs for 30 min at 4 °C, then fixed and permeabilized by using the BD Cytofix/cytoperm Plus Kit (BD Biosciences, San Jose, CA, USA) and stained for intracellular interferon γ (IFN-γ) for 30 min at 4 °C. The following Abs were used: APC Alexa Fluor 750-conjugated anti-CD16 (clone 3G8), ECD-conjugated anti-CD56 (clone N901), PE-conjugated anti-IFN-γ (clone 45.15) and isotype control (Beckman Coulter, CA, USA), PC5-conjugated anti-CD107a (clone H4A3) and isotype control (BD Biosciences). Functional responses and MFI of each fluorochrome of cell subsets were analyzed by FCM.

### 2.6. Statistics

All data are presented as mean ± SD unless otherwise stated. Statistical and graphical analyses involved using Graphpad Prism (GraphPad Software, San Diego, CA, USA). Statistical significance was determined by one-way ANOVA to compare differences among multiple groups. *p* < 0.05 was considered statistically significant.

## 3. Results

### 3.1. Production and Physiochemical Characterization of the Constructs

**Figure 1:**
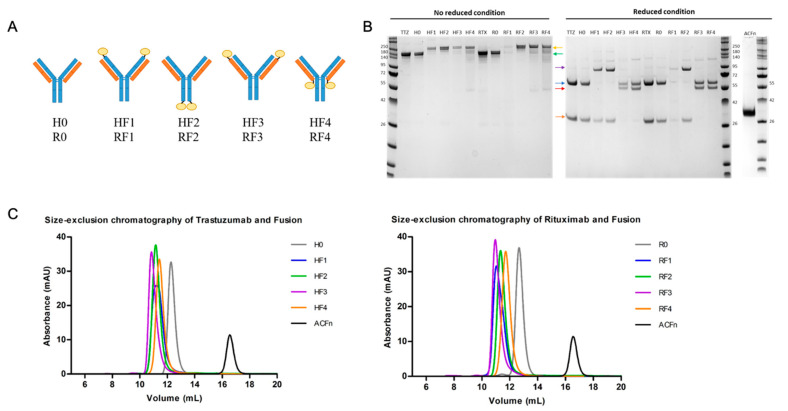
(**A**) Cartoon representations of the 8 BsAbs. HF and RF families were constructed on trastuzumab (TTZ) and rituximax (RTX) commercial therapeutic mAbs and produced as biosimilars, as H0 and R0, respectively. Anticalin ACFn (yellow) was covalently linked to heavy (blue) or light (orange) chains by using Glycine-Serine linkers at N- and C-terminal positions. (**B**) SDS-PAGE of the mAbs and BsAbs in non-reducing and reducing conditions; ACFn is the anti-fibronectin anticalin. Green and yellow arrows indicate mAbs and BsAbs, respectively, in the non-reduced condition. In the reduced condition, heavy chains are indicted by blue or purple arrows and light chains by orange or red arrows for wild-type chains or chains linked to anticalin, respectively. (**C**) Size-exclusion chromatography on a calibrated Superdex 200 Increase 10/300 GL column of each antibody and the anticalin (right trastuzumab and BsAb, left rituximab and BsAb).

The different molecules were constructed with an anticalin molecule fused to the C- or N-terminal of heavy (H) and light (L) chains of TTZ (H) and RTX (R) by a peptide linker (Figure S1). The nomenclature applied indicates the position of the anticalin on the Ab by a number according to the diagram in Figure 1A. Positions 1 and 2 correspond to the N- and C-terminals on the H chain and positions 3 and 4 to the N- and C-terminals on the L chain. The number 0 indicates the absence of anticalin on the mAb.

All BsAb productions were analyzed by SDS-PAGE to confirm their integrity in non-reducing or reducing conditions. H0 and R0 had the same profile as the corresponding commercial mAb (Figure 1B). Non-reduced Abs appeared at about 150 kDa and the H and L chains as expected, at 50 kDa and 25 kDa, respectively, in the reduced condition. All BsAbs had higher mass due to the presence of anticalin. In the no-reduced condition, Abs appeared at about 170 kDa. In the reduced condition, when anticalin was on the H chain (positions 1 and 2), the mass increased to 75 kDa. Likewise, when anticalin was on the L chain (positions 3 and 4), the mass increased to 50 kDa (Figure 1B).

Further analyses with size-exclusion chromatography (SEC) showed mostly the monomeric form for all mAbs and BsAbs (Figure 1C and Figure S2). The yield seemed to vary more with the origin of the mAb than the position of the anticalin (Table 1). Fusion of anticalin with TTZ tended to decrease yield by 40%, with a slightly greater effect when the anticalin was on the C-terminal (positions 2 and 4). With RTX, which is slightly less well expressed, only the yield of RF3 decreased. After purification, a buffer change was performed from citrate pH 3.0 to PBS. Aggregation resulting in loss of mAbs and BsAbs was always observed. This loss appeared to be less for HF4. However, the anticalin in position 3 (for H and R) seemed to cause instability with the pH change (Table 1). The stability of molecules analyzed by Prometheus showed that the fusion temperatures were not really affected by the presence of anticalin (Figure S3). The thermic profile of denaturation and the mean of aggregation temperature for all mAbs and BsAbs were both non-significantly different and quite similar to their respective references (H0/R0), except for both forms on position 3, whose temperature of aggregation was reduced (Figures S4–S7). A longer time to analyze stability did not show any difference (Figures S4–S7).

**Figure 2:**
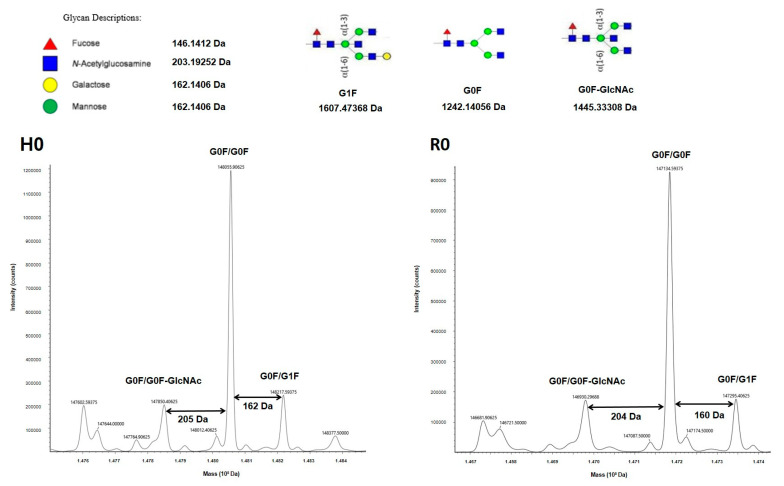
Mass spectra of H0 and R0 monoclonal Abs acquired under denaturing conditions. Insets correspond to the glycoforms identified.

**Figure 3:**
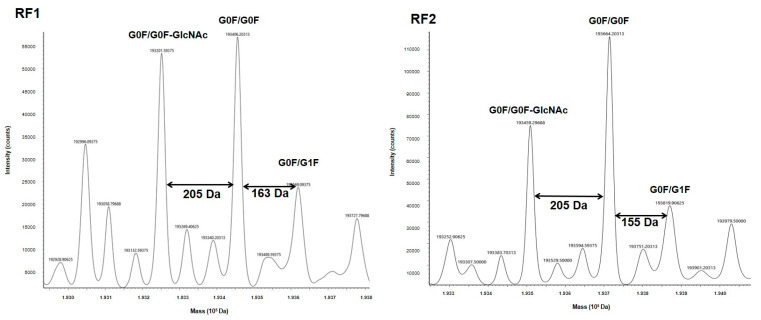

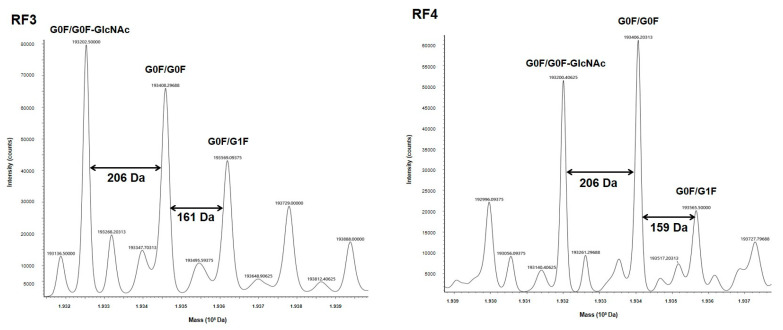
Mass spectra of BsAbs of RF format.

Because mAb glycosylation is a major property of the Ab structure and therapeutic effects, we analyzed the mass of each mAb and BsAb to determine their glycosylation. First, H0 and R0 were analyzed, and the main glycoforms observed were G0F/G0F, G0F/G0F-GlcNAc and G0F/G1F (Figure 2). The mass spectra of the RF1-4 (Figure 3) and HF1-4 (Figure S8) BsAbs showed the same glycoforms as those identified for the native mAbs R0 and H0.

### 3.2. Binding of the Constructs to Their Antigen and Anticalin Target

**Figure 4:**
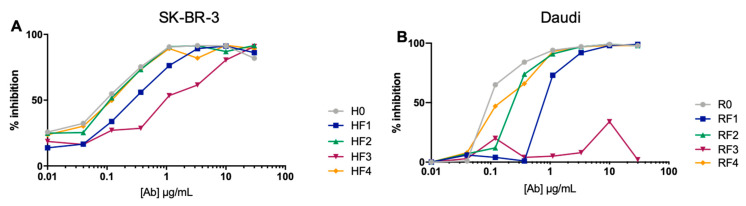
Antigen recognition by BsAbs. Competition assays using (**A**) fluorescent trastuzumab (TTZ-^A488^) or (**B**) rituxumab (RTX^-A488^) on SK-Br-3 or Daudi cell lines, respectively. The mean fluorescent intensity (MFI) of TTZ^-A488^ and RTX^-A488^ were determined by flow cytometry in the presence of mAbs and BsAbs at different concentrations. Data are percentage TTZ^-A488^ inhibition binding on SK-Br3 cells or RTX-^A488^ inhibition binding on Daudi cells. RTX^-A488^ or TTZ^-A488^ alone was considered 100% binding.

We evaluated the capacity of constructs to bind to antigenic targets by a competition assay on SK-BR-3 (expressing Her2) and Daudi (expressing CD20) cell lines. Each sample of trastuzumab^AF488^ or rituximab^AF488^ was first evaluated alone on their respective cells dose-dependently to calculate the appropriate amount of mAb necessary to obtain 100% fixation. The results are presented in Figure 4 as the percentage inhibition of trastuzumab^AF488^ or rituximab^AF488^ binding. As expected, H0 and R0 exhibited dose-dependent inhibition as did all BsAbs with anticalin at positions 1, 2 and 4. HF3 exhibited slightly lower inhibition, whereas RF3 inhibition was greatly reduced, thus indicating lower binding or near absence of binding of these BsAbs to their respective antigens. This lower affinity of HF3 did not appear on the values of K_D_ calculated for H0 and HF1-4 family against Her2 by BLI (Figure S9A, Table S1).

The FN binding was evaluated by measuring K_D_ calculated by BLI with recombinant FN-Hist docked on anti-Hist sensors (Figure S9B). The results summarized in Table 2 show moderately reduced values of KD for BsAbs of the HF family (except for HF3:1-log less) compared with ACFn (KD close to 10^−6^). BsAbs of the RF family showed a different situation: the affinity of the four BsAbs for Fn was more substantially decreased: at least 1-log less for RF3 and RF 4 and 2-log less (K_D_ close to 10^−6^) for RF1 and RF2 compared with ACFn, respectively.

### 3.3. Binding of Constructs to Fcγ Receptors and Fc-Dependent Functional Activation

**Figure 5:**
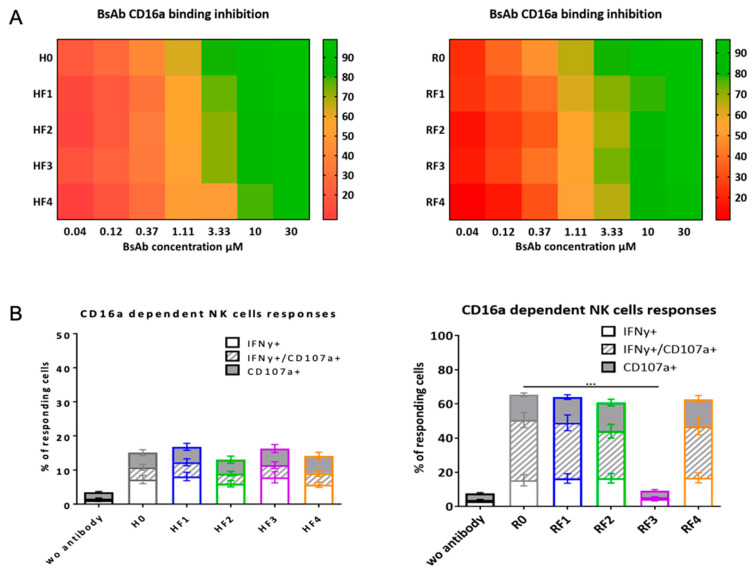
BsAbs binding to CD16 (FcγRIIIA). (**A**) Heatmap representation of mAb and BsAb binding to CD16 evaluated by a flow cytometry competition assay of anti-CD16-FITC binding on NK92 hCD16V in the presence of mAbs or BsAbs for 30 min at 4 °C. Data are percentage inhibition binding of anti-CD16-FITC. (**B**) CD16-dependent BsAb functionality with NK92 hCD16V cells as effector cells and SK-Br-3 or Daudi cells as target cells in the presence of mAbs or BsAbs. NK92 cells were labelled with CD107a, CD16 and CD56 Abs, then intracellularly labelled for INF-γ. Data are percentage NK92 cells expressing CD107a and/or INF-γ. (One way Anova, *** *p* < 0.005).

To evaluate construct binding on the CD16A receptor (FcγRIIIA), we used a competition assay with NK92 hCD16V cells and fluorescent anti-CD16A and flow cytometry. The results are expressed as percentage inhibition of binding of anti-CD16A fluorescent mAbs evaluated as 100% binding without competitor Abs. Results are represented as a heatmap (red for the lowest values and green for the highest) in Figure 5A. All BsAbs were able to inhibit the anti-CD16A mAb fixation, but HF4, HF3 and HF2 had slightly lower capacity than H0 at 3.33 μM. RF4, RF1 and RF2 exhibited slightly lower capacity of anti-CD16A inhibition than R0 at the same concentration.

The capacity of BsAbs to engage a functional CD16A-dependent response was evaluated with NK92hCD16V cells. The activation of NK92 hCD16V cells was measured with the expression of membranous CD107 and the intracellular presence of INF-γ in the presence of SK-Br3 or Daudi cells and mAbs or BsAbs. The BsAbs of the HF family exhibited low activation (<20%) with HF2, which had the lowest value (Figure 5B). The activation levels of NK92 hCD16V with R0, RF1, RF2 and RF4 were substantial and similar, whereas RF3 did not induced a significant response (*p* < 0.005).

**Figure 6:**
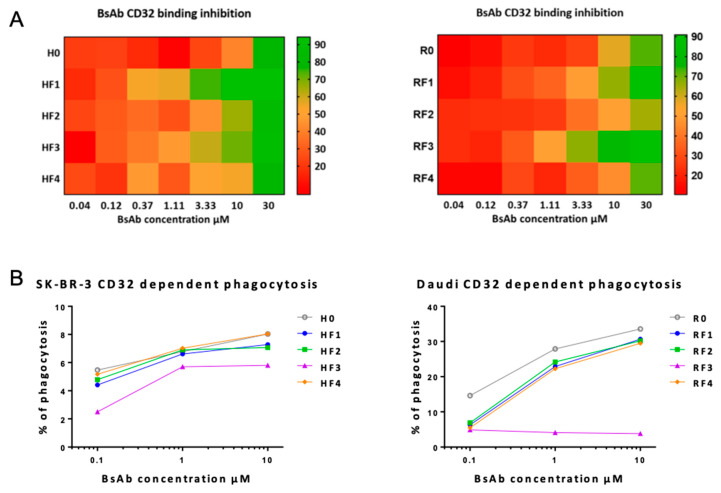
BsAbs binding to CD32 (FcγRIIA). (**A**) Heatmap representation of BsAbs binding to CD32 evaluated by a flow cytometry competition assay of anti-CD32-FITC binding on THP1 cells in the presence of BsAbs. Data are percentage binding inhibition of anti-CD32-FITC. (**B**) Phagocytosis assays were used to evaluate CD32-dependent BsAb functionality. Data are percentage of fluorescent positive cells. Results are represented as described in Figure 5.

To study CD32 (FcγRIIA) binding, we used a competition assay with THP1 cells incubated with Mabs or BsAbs and FITC-conjugated anti-CD32A and flow cytometry. All members of the HF family were able to compete with anti-CD32A fluorescent mAbs, but HF1 and to a lesser extent HF3 exhibited higher binding inhibition (Figure 6). For the RF family, RF3 and to a lesser extent RF1 were the most efficient in inhibiting the binding of the fluorescent mAb to CD32.

The functional capacity of BsAbs to functionally engage CD32A was established with a phagocytic assay using monocyte-induced macrophages and CFDA-SE–labeled SKBR3 or Daudi cells as a target (Figure 6B). The ability of BsAbs from the HF family to induce phagocytosis was relatively weak (<15%) and similar whatever the anticalin position. For RF family BsAbs, the percentage of phagocytosis was higher with R0 (up to 30% at 10 μL/mL) than with RF1, RF2 and RF4 (about 15% at 10 μL/mL) but was undetectable with RF3 (whatever the concentration).

### 3.4. Binding of Constructs to FcRn and FcRn-Dependent Transcytosis

**Figure 7:**
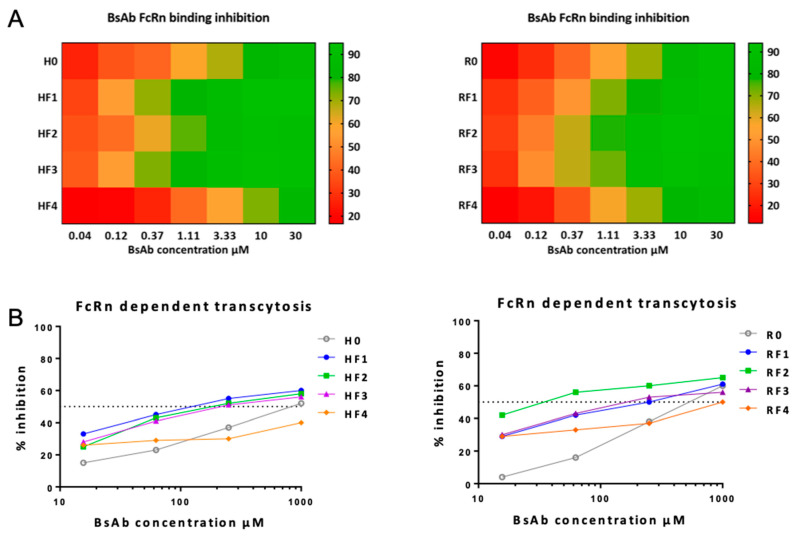
BsAbs binding to FcRn. (**A**) Heatmap representation of BsAbs binding to FcRn evaluated by flow cytometry in a competition assay of rituxumab^AF488^ on a mix of Jurkat and JurkatΔFcRn cells (ratio 1:1) in the presence of mAbs or BsAbs at different concentrations. Data are percentage inhibition binding of rituxumab^AF488^. (**B**) FcRn-dependent BsAb functionality was evaluated by a transcytosis assay with MDCKII cells expressing human FcRn in the presence of mbs or BsAbs. Rituxumab^-AF488^ fluorescence in the compartment receiving transcytosed Rituxumab^-AF488^ was measured by microplate reader and data are percentage inhibition of rituximab^AF488^ transcytosis. Results are represented as described in Figure 5 and Figure 6.

To evaluate the FcRn binding of mAbs and BsAbs, we used a competition assay with FcRn transfected Jurkat cells and rituximab^AF488^ in the presence of the different BsAbs (Figure 7A). All BsAbs from both RF and HF families were able to bind to FcRn. However, the anticalin in position 4 did not modify the affinity to FcRn as compared with that observed without anticalin (HF4 vs. H0 and RF4 vs. R0). By contrast, the presence of anticalin in position 1 (especially for HF1), 2 (especially for RF2) and 3 (both HF3 and RF3) substantially increased the binding to FcRn.

To evaluate the functional FcRn-dependent activity of these BsAbs, we performed a transcytosis assay with MDCKII cells expressing human FcRn. Rituximab^AF488^ transcytosis was measured with a spectrofluorometer in the presence of the different mAbs or BsAbs. Results are expressed as percentage transcytosis inhibition considering the transcytosis of rituximab^AF488^ alone as 100% (Figure 7B). All BsAbs were able to inhibit the transcytosis dose-dependently. For both HF and RF families, almost all BsAbs with anticalin in positions 1, 2 and 3 were better inhibitors than mAbs without anticalin or BsAbs with anticalin in position 4.

## 4. Discussion

In this work, we evaluated the different positions of an anticalin on Ab shape to analyze the advantages of this BsAb format in terms of simplicity of construction and modularity of the different partners. We associated two different Ab shapes, TTZ and RTX, for this anticalin because both Abs are well characterized and tools to analyze their functionality are available.

TTZ is a therapeutic mAb inhibiting Her2 dimerization and blocking tumor cell proliferation [16]. Competition assays and ELISA using Her2-expressing cells and recombinant Her2 are the best way to evaluate this binding affinity [17]. In contrast, RTX is a mAb used for many B-cell malignancies with high response and long-term remission [18]. Affinity analyses of RTX and derived molecules are complex because CD20 antigen is expressed on the surface in a supramolecular form [19]. Therefore, in this case, competition assays with fluorescent antibody and CD20-expressing cells are the easiest way to compare the BsAb binding to CD20.

Today, predicting the production of recombinant mAbs is not possible, although some in silico tools have been developed for this purpose [20]. H0 corresponds to trastuzumab. As well, the R0, biosimilar of RTX and the anticalin N7A (ACFn) have been a natural and necessary basis for this work. H0 appears to have better production than R0. Under the same production conditions, anticalin has a yield of 30 mg/L (Table 1). This yield in milligrams per liter, or even converted to millimoles per liter, is still lower than that of R0 and H0. However, regardless of its position, it has little effect on the production of RF BsAbs, although the N-terminal is considered important for production. ACFn seems to have more effect on HF BSAb production. Comparing the production yield of these two families of Abs (HF and RF) seems to indicate the primacy of the mAb structure, which affects the production yield more than the position of anticalins on it. In any case, all BsAbs could be obtained in sufficient quantities for all analyses.

The anticalin does not interfere with the structure of the mAbs as seen by SDS-PAGE under non-reducing conditions. Its presence does not alter the oligomeric profile of the Abs. However, the elution volume is a function of the location, with greater impact for positions 3, 1, 2 and 4 (i.e., when the anticalin is placed toward the paratope of the Ab). The conformation can also be studied by thermal profile analysis. Here again the absence of significant difference does not allow drawing a real conclusion. The anticalin has a Tm of 64.5 °C, different from the TM1 of H and R, but regardless, there is no marked trace of the anticalin at any position on the BsAb profile.

In terms of aggregation temperature, with position 3, both BsAbs (RF3 and HF3) had a similar temperature to that of anticalin alone. On close observation, the curves show two aggregation states: that of the anticalin and that of the mAb. Thus, at position 3, the anticalin seems to be able to aggregate alone before the mAb, whereas at the other three positions, it seems to be protected, probably showing a stronger interaction with the mAb domains than at the hinge region.

Higher-order structure and glycosylation profile of a therapeutic mAb are important determinants of its function. Both TTZ and RTX, as for IgG1, carry N-glycosylation on Asn 297, which is essential for stability and functional activities [21,22,23,24]. MS is a valuable tool for both structure and glycosylation validations for our constructs. MS profiles of both mAbs revealed at least three major glycoforms (G0/G0F, G0F/G1F, G0/G0F-GlcNAC), which are observed on both commercial and biosimilar molecules [25,26,27]. In fact, MS also confirmed that the glycosylation level was similar between all BsAbs and their respective H0 and R0 counterparts. Especially, the fucosylation level, which has a huge impact on functional properties [28], was similar to that observed for H0 and R0. So, all the constructs conformed to what was expected and represented valuable BsAbs.

**Figure 8:**
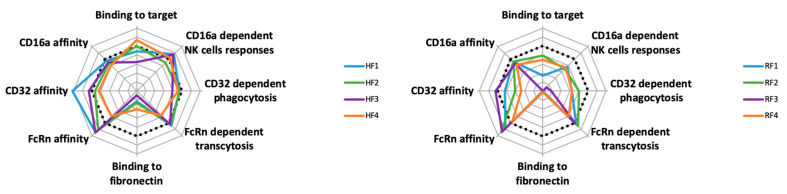
Spider representation of BsAb properties. Molecules H0, R0 and ACFn were considered as 100% functionality represented by the dotted line.

Overall, the properties of both HF and RF families are not superimposable, even concerning yield (Figure 8). Some BsAbs, such as RF3, showed no functional activities. However, some, such as HF1, seemed to have improved functionality. The carrying mAb affected both BsAbs and their functionality, and according to the mAb, the impaired formats differ: RF3 for the RF family and HF4 for the HF family. Thus, BsAb functionalities seemed to depend on the chosen mAb. Second, within each family, no remarkable BsAb stands out, except for HF4/RF4, which demonstrated lower affinity for CD16A and CD32A (more so RF4) (Figure 8). Except for RF3, the binding of each Ab to its antigenic target was slightly modified. This latter position with ACFn in the N-terminal of the L chain, which impairs the mAb binding probably for stereotypic reasons, could also occur for position 1, yet HF1 and less so RF1 had standard binding to their antigen. This reinforces the importance of the carrier mAb on the BsAb behavior.

Anticalin binding to Fn was variably impaired (Table 2, Figure S9 and Figure 8), more so for the RF than HF family for mAb-linked anticalin. Concerning the RF family, its global Fn affinity was much lower than free ACFn, and RF1, with 200-times less affinity as the worst position for Fn binding. In this latter case, the Fn binding site could be hidden because of the different possible conformations of ACFn at the end of its arm. Interactions of ACFn with the Ab structure could depend on the size and rigidity of the arm, its conformation and interactions on the domains of the mAb. Some other anticalins must be tested in these different configurations to evaluate the relative impact of the anticalin affinity versus the carrying mAb.

In contrast, the presence of ACFn has various effects on the FcR affinities of BsAbs: it seemed to have little effect on FcγRIIIA/CD16A binding and improving FcRn binding and had a mixed effect on FcγRIIA/CD32A binding. For this latter binding, positions 2 and 4 for both BsAb families seemed less favorable. Access to the hinge-CH2 region is highly important for CD32A binding; both positions were closest to this region, which might impede this FcR binding. Nevertheless, except for RF3, functional activities depending on FcγR binding seem to be less affected by the presence of ACFn. However, the binding to the antigenic target seemed the most important event, whether with NK92 activation or phagocytosis functions, as shown by the fact that both functions were impossible for RF3, which did not bind to CD20. Another important parameter in the study of new format BsAbs, is the binding of FcRn, because it is a key molecule involved in IgG and albumin recycling and transcytosis [29]. Via cellular transcytosis, it is responsible for the two ligands, allowing their biodistribution all over the body [30]. Hence, it is involved in pharmacokinetics and pharmacodynamics of therapeutic molecules bearing an Fc portion, and many studies deal with these properties to extend the FcRn-dependent half-life of these biotherapies [31]. Binding of IgG to FcRn occurs at acidic pH, whereas it is extremely low at neutral pH and involves the CH2-CH3 domains with the participation of histidine [32]. In this work, we found no significant difference in binding and consequently transcytosis of BsAbs of the HF and RF families with anticalin at position 1 to 3. Of note, no position compromised the link to FcRn; all were better (position 1 to 3) or the same (position 4) as the related mAbs. Therefore, positions 1 to 3 had a positive effect on binding and transcytosis of BsAbs of the HF family as compared with H0 and HF4 and of the BsAbs of the RF family as compared with R0 and RF4. Different teams have published that mutations located at a distance from the FcRn binding site may modify IgG affinity for the receptor [15,33]. Isoelectric-point and charge modifications may explain this difference. Based on our results, it is difficult to draw a rule relating distance and affinity for FcRn. Indeed, the BsAbs with ACFn in position 1 or 3 (i.e., located very far from the binding site in the Fab) and 2 (located close to the FcRn biding site in the Fc) had similarly high affinity, although the profile also depended on the family (HF1 = HF3 > HF2 and RF1 < RF2 = RF3). By contrast, the affinity of the BsAbs with AFCn in position 2 and 4, which are on either side of the FcRn binding site and at a close distance to it, were substantially different (HF2 >> HF4 and RF2 >> RF4).

Until now, at least two BsAbs involving anticalins have been developed, including anti-Her2 Abs associated with anticalin anti-CD137 [13] or anti-PD-L1 associated with anticalin anti-4-1BB [34]. In both cases, the anticalin was merged on the C-terminal of the H chain (position 2), but no other position has been tested. Our work aimed to provide an overview of all possible positions from the perspective of BsAb production, binding properties and functionalities. In light of our data, position 2 seems a reasonable choice because all binding or functional properties were maintained but this could depend on the mAb choice. Likewise, position 4 should be avoided because FcRn-dependent homeostasis may be compromised, but this point may depend on the carrying Ab also.

In summary, the findings of this work are an important demonstration that BsAbs with linked anticalin are an easy and safe way to build new BsAbs that could be adapted to each therapeutic need. Nevertheless, as for all mAbs, the BsAb structural configuration can affect functionalities and elimination differently. Careful evaluation of each position of anticalin linking might be necessary to lead to the selection and design of BsAbs with optimized therapeutic values.

## Figures and Tables

**Table 1:** Average yields and losses for constructs.

Constructs	Yield ^1^ (mg/mL)	Yield Ratio	Loss ^2^ (%)
H0	525.5 ± 382	1	22.7 ± 8.3
HF1	354.6 ± 125	0.68	22.2 ± 11.6
HF2	291.3 ± 122	0.55	19.0 ± 9.4
HF3	373.2 ± 163	0.71	27.7 ± 13.9
HF4	257.1 ±175	0.49	9.5 ± 4.0
R0	299.5 ± 122	1	20.1 ± 10.2
RF1	296.5 ± 92	0.99	18.8 ± 9.7
RF2	265.5 ± 124	0.89	18.5 ± 12.9
RF3	216.2 ± 80	0.72	32.6 ± 13.6
RF4	284.7 ± 68	0.95	20.9 ± 10.81
ACFn	30 ± 5	-	19.6 ± 5.74

Data are mean ± SD. ^1^ Yield was calculated by the concentration of purified protein (mg/mL)/total volume of production). ^2^ Loss was calculated as (100 minus % of purified protein/total concentration protein).

**Table 2:** K_D_ values of BsAbs against fibronectin.

Constructs	K_D_^1^	Ratio H-RF/ACFn ^2^
HF1	8.64 × 10^−8^ ± 1.14 × 10^−9^	4.48
HF2	7.57 × 10^−8^ ± 1.17 × 10^−9^	3.92
HF3	1.99 × 10^−7^ ± 4.81 × 10^−9^	10.33
HF4	4.77 × 10^−8^ ± 4.23 × 10^−^^10^	2.47
RF1	4.99 × 10^−6^ ± 3.63 × 10^−^^6^	258.55
RF2	1.28 × 10^−6^ ± 3.92 × 10^−^^7^	66.48
RF3	8.09 × 10^−7^ ± 1.53 × 10^−^^7^	41.93
RF4	8.87 × 10^−7^ ± 1.44 × 10^−^^7^	45.96
ACFn	1.93 × 10^−8^ ± 1.65 × 10^−^^10^	1

Data are mean ± SD unless indicated. ^1^ K_D_ calculated by BLI, mean ± SD of 3 independent experiments. ^2^ ratio of K_D_ value of HF or RF to that of ACFn.

## Data Availability

Data may be made available upon request.

## Supplementary Materials

The following supporting information can be downloaded at: https://www.mdpi.com/article/10.3390/antib11030054/s1, Figure S1: Scheme of plasmids construction; Figure S2: Percentage of monomeric form and elution volume for each protein after analysis of size-exclusion chromatography. Figure S3: Profiles of curves for Prometheus analyses; Figure S4: Kinetics of protein stability of HF BsAb; Figure S5: Thermal and aggregation analysis by Prometheus for TTZ and BsAbs (HF1-HF4); Figure S6: Kinetics of protein stability of RF BsAbs; Figure S7: Thermal and aggregation analysis by Prometheus for RTX and related BsAbs (RF1-RF4); Figure S8: Mass spectra of HF BsAbs (H1-H4); Figure S9: sensorgrams of both HF1-4 and RF1-4 against Fn, and H0 and HF1-4 against Her2; Table S1: K_D_ values of HF family against Her2.
