# Supplementary material for: Anticalin N- or C-Terminal on a Monoclonal Antibody Affects Both Production and In Vitro Functionality
Source: Antibodies (Basel). 2022 Aug 22;11(3):54. doi: 10.3390/antib11030054 (PMC9397084; doi:10.3390/antib11030054)
Supplement: Supplementary file 1 [file antibodies-11-00054-s001.zip › antibodies-1813240-supplementary.pdf]

Supplementary Table

Table S1. K_D values of HF family against Her2

**Constructs**	**KD (M) ± SD**[1]
H0	7.11E-09 ± 1.04E-08
HF1	5.81E-09 ± 6.85E-09
HF2	1.40E-09 ± 8.46E-10
HF3	3.88E-09 ± 4.83E-09
HF4	2.13E-09 ± 1.61E-09

Data are mean±SD unless indicated

[1]*K_D calculated by BLI at 25°C. Mean of 5 independent experiments.*

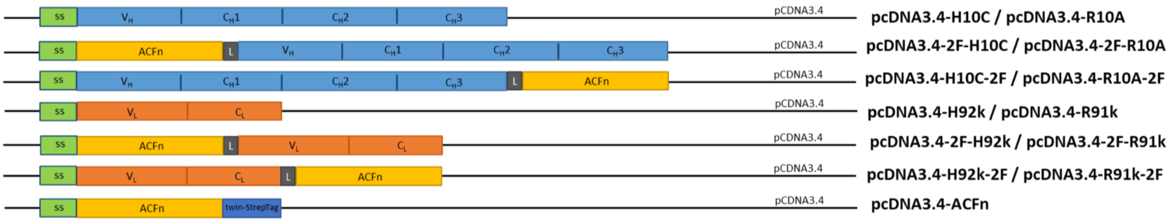

Figure S1: Scheme of plasmids construction needed for both Ab and BsAb production

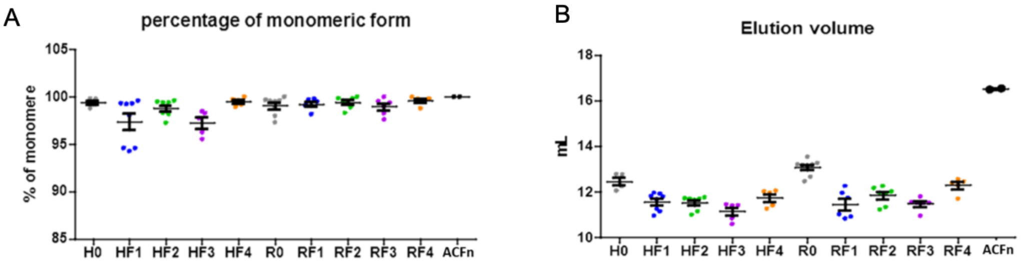

Figure S2: (**A**) Percentage of monomeric form and (**B**) elution volume for each protein after analysis of size-exclusion chromatography for each production

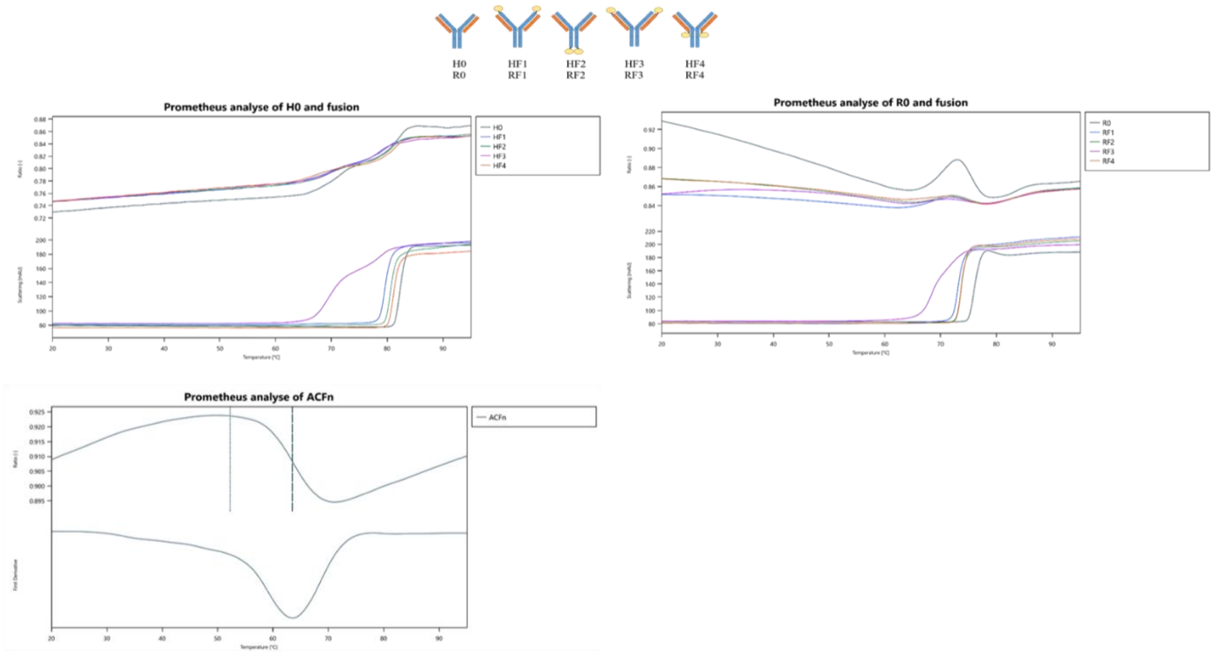

Figure S3: Profile of curves obtained by Prometheus for nanoDSF analyse

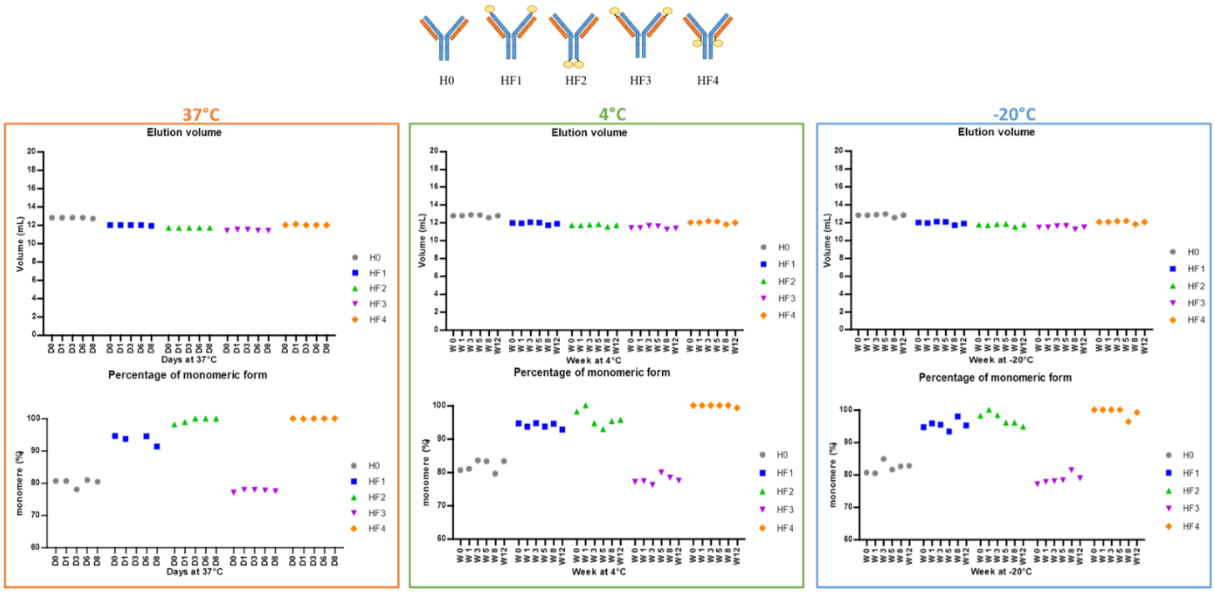

Figure S4: Kinetics of protein stability during storage for height days at 37°C or twelve weeks at +4°C or -20°C. Analyze by size-exclusion chromatography on a calibrated Superdex 200 10/300 GL column of elution volume and percentage of monomeric form of each antibody for Trastuzumab and related BsAb (HF1-HF4).

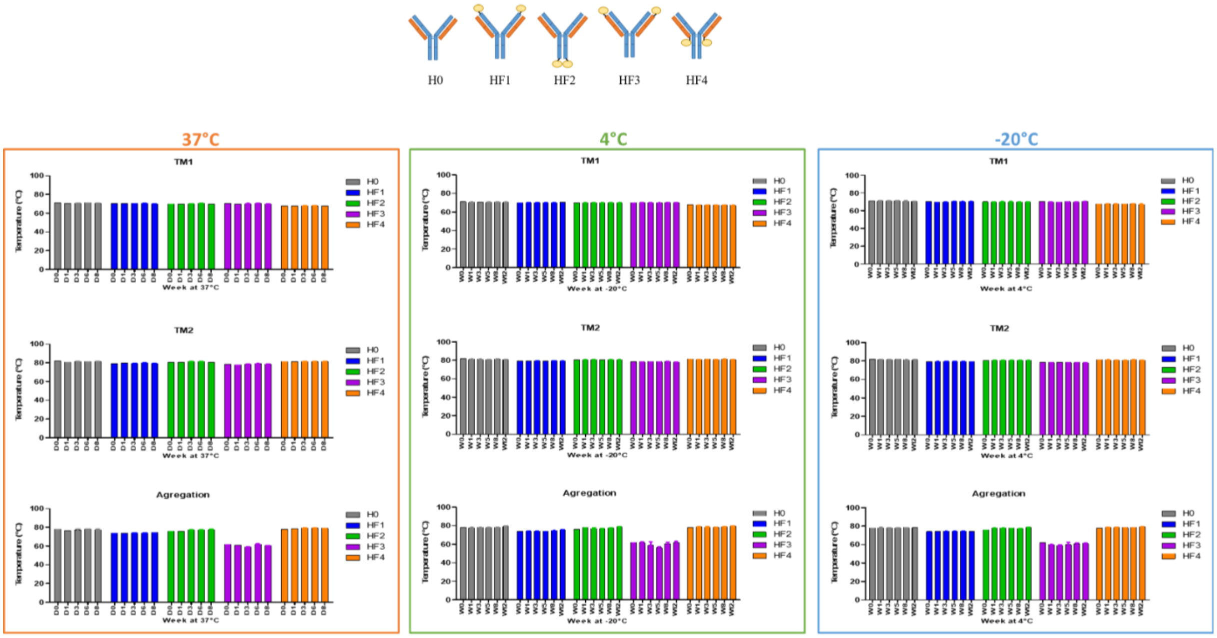

Figure S5: Kinetics of protein stability during storage for height days at 37°C or twelve weeks at +4°C or -20°C. Thermal and aggregation analysis by Prometheus for Trastuzumab and BsAb (HF1-HF4).

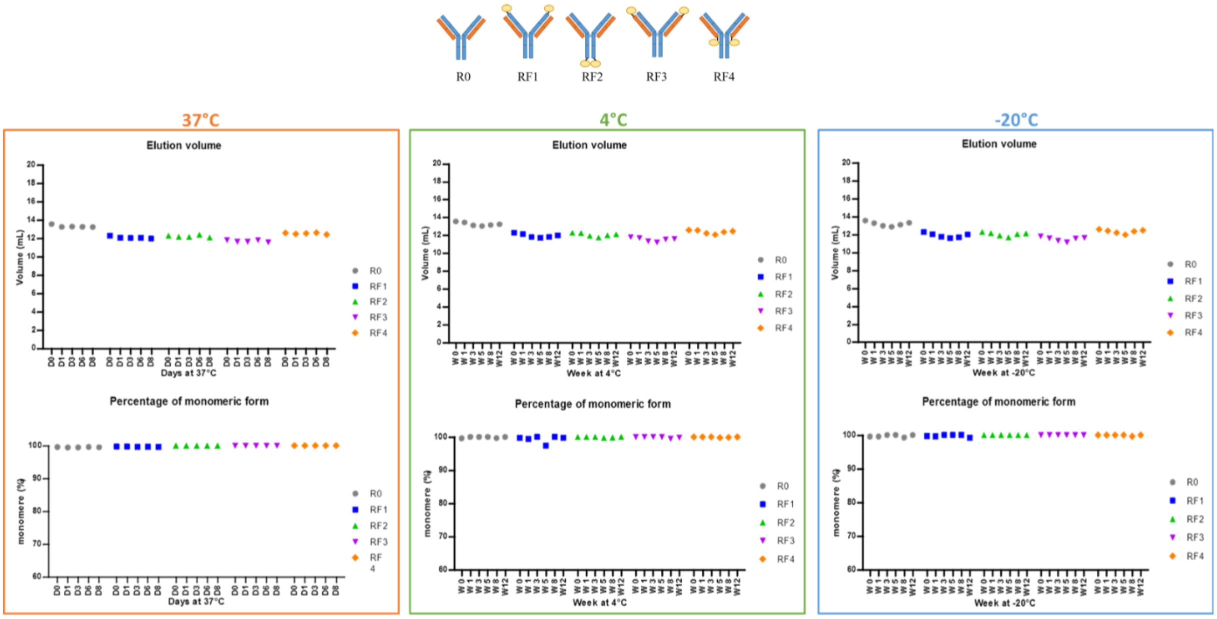

Figure S6: Kinetics of protein stability during storage for height days at 37°C or twelve weeks at +4°C or -20°C. Analyze by size-exclusion chromatography on a calibrated Superdex 200 10/300 GL column of elution volume and percentage of monomeric form of each antibody for Rituximab and related BsAb (RF1-RF4).

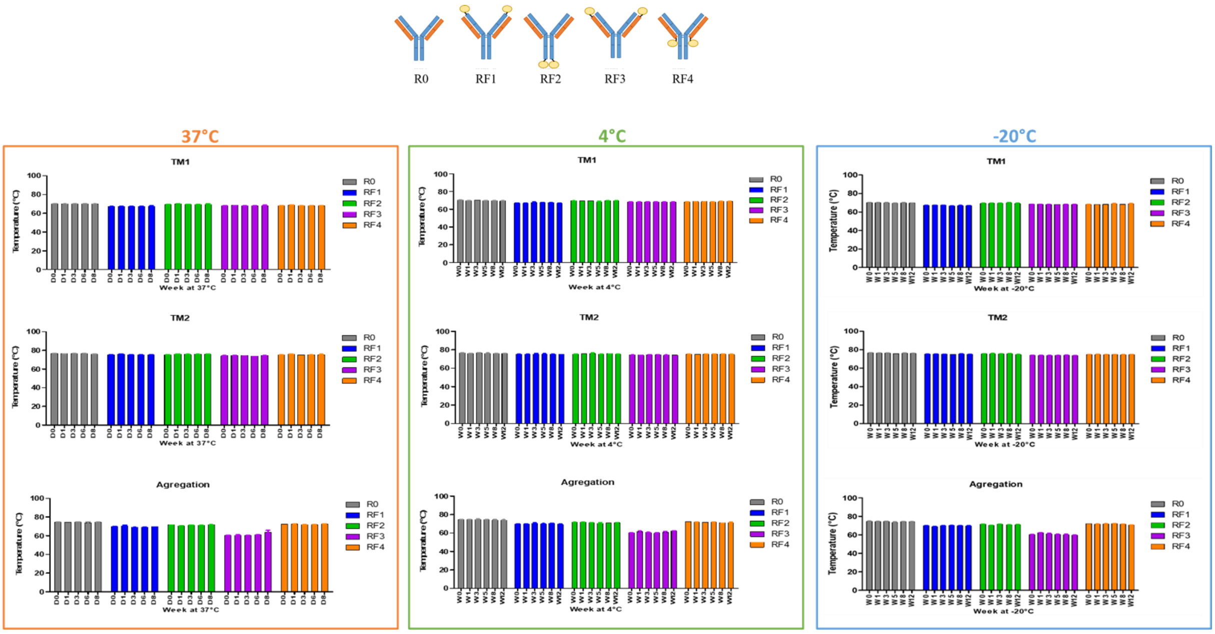

Figure S7: Kinetics of protein stability during storage for height days at 37°C or twelve weeks at +4°C or -20°C . Thermal and aggregation analysis by Prometheus for Rituximab and related BsAb (RF1-RF4)

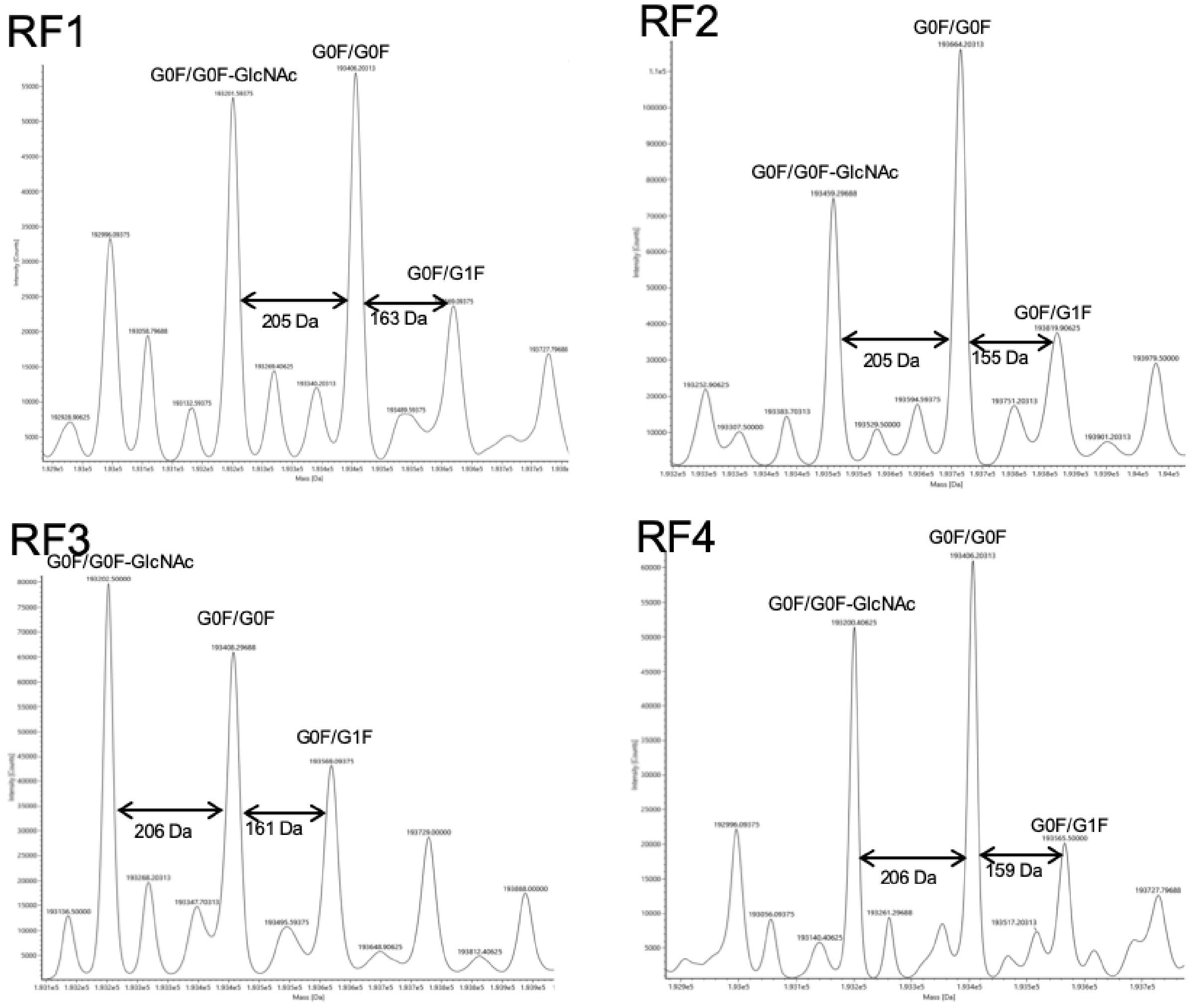

Figure S8: Mass spectra of HF BsAb

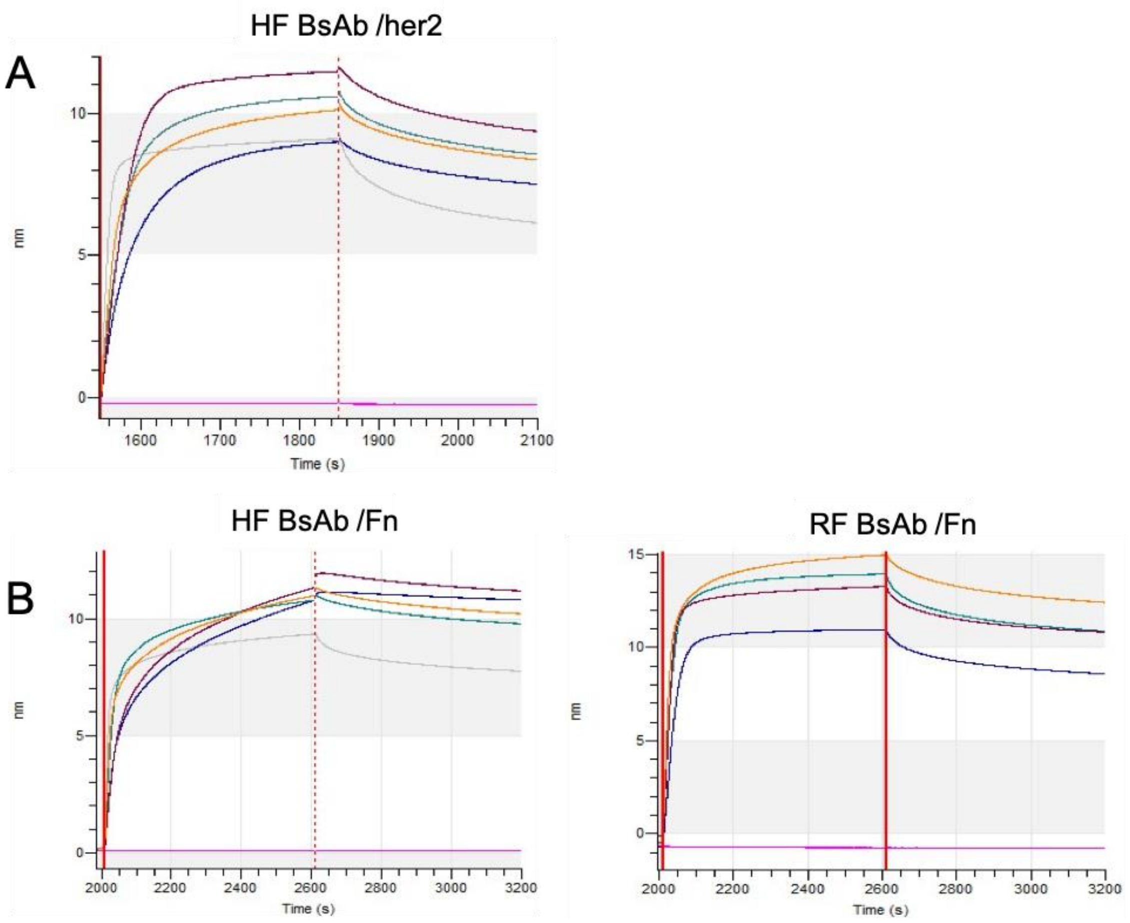

Figure S9: Sensorgrams of (A) H0-H4 at 200 nM on Her2, (B) H0-H4 and R0-RF4 at 200 nM or Fn obtained by BLI. H0/R0 in gray, HF1/RF1 in blue, HF2/RF2 in green, HF3/RF3 in red, HF4/RF4 in orange, buffer in pink.
